# Excited‐State Dynamics of a Two‐Photon‐Activatable Ruthenium Prodrug

**DOI:** 10.1002/cphc.201501075

**Published:** 2016-01-06

**Authors:** Simon E. Greenough, Michael D. Horbury, Nichola A. Smith, Peter J. Sadler, Martin J. Paterson, Vasilios G. Stavros

**Affiliations:** ^1^ Department of ChemistryUniversity of Warwick, Gibbet Hill RoadCoventryCV4 7ALUK; ^2^ Institute of Chemical SciencesHeriot-Watt UniversityEdinburghEH14 4ASUK; ^3^ Department of ChemistryUniversity of SheffieldSheffieldS3 7HFUK

**Keywords:** density functional calculations, ligand substitution, ruthenium, time-resolved spectroscopy, two-photon absorption

## Abstract

We present a new approach to investigate how the photodynamics of an octahedral ruthenium(II) complex activated through two‐photon absorption (TPA) differ from the equivalent complex activated through one‐photon absorption (OPA). We photoactivated a Ru^II^ polypyridyl complex containing bioactive monodentate ligands in the photodynamic therapy window (620–1000 nm) by using TPA and used transient UV/Vis absorption spectroscopy to elucidate its reaction pathways. Density functional calculations allowed us to identify the nature of the initially populated states and kinetic analysis recovers a photoactivation lifetime of approximately 100 ps. The dynamics displayed following TPA or OPA are identical, showing that TPA prodrug design may use knowledge gathered from the more numerous and easily conducted OPA studies.

Ruthenium pyridyl complexes have been deployed in a myriad of technological and medical applications, such as light harvesting,^1^ light‐emitting devices,^2^ fluorescence imaging,^3^ cytotoxic action,3b, 4 and, of particular relevance to the present study, photodynamic therapy (PDT).^5^, ^6^ In PDT, an inert precursor drug is activated with light. The afforded spatial control limits possible side effects to the immediate area of irradiated tissue,^7^ and has the potential to generate unique reactive species that might otherwise be biologically incompatible, that is, caged delivery.^8^ Although PDT is now used to treat a number of skin conditions,^9^ a major hindrance to its more widespread usage is the low transmittance of UV and visible light through biological tissue, with a transmission, or PDT window, existing between 620 and 1000 nm.^10^

To circumvent the absorption of UV/Vis radiation by tissue, two‐photon absorption (TPA) has been utilized over one‐photon absorption (OPA),^11^ in which the precursor drug is now activated by radiation in the PDT window in contrast to UV/Vis radiation (OPA). From a precursor drug design point of view, it is imperative to understand the initial activation mechanism following TPA for such species. Such knowledge will provide a platform for the future design of more effective PDT agents (e.g. optimum choice of substituents).^12^ To address the apparent paucity of the mechanistic insight following TPA, we studied a ruthenium(II) complex containing nicotinamide, a water soluble vitamin (part of the vitamin B group), *cis*‐[Ru(bpy)_2_(NA)_2_]^2+^ (**1**, shown in Figure 1 inset) (bpy=2,2′‐bipyridine and NA=nicotinamide, pyridine‐3‐carboxamide). Following excitation of **1** with UV/Vis light (i.e. OPA), it is well established that the initial photoactivation mechanism involves the formation of the mono‐aquated species [Ru(bpy)_2_(NA)(H_2_O)]^2+^ (**2**), as shown by the UV/Vis absorption spectra in Figure 1. This transformation occurs on a picosecond (ps) timescale, and is mediated via a pentacoordinate intermediary species.^12^ To allow the activation of **1** in the PDT window, we used TPA with 800 nm radiation. The present study addresses whether there are significant differences between the one‐ and two‐photon activation mechanisms of **1** by tracking the dynamics of photoactivation following TPA. Particularly, it is the role of the excited‐state deactivation pathways in TPA and OPA, that is, those competing with the formation of **2**, that we are interested in examining. The main processes of concern are internal conversion of the lowest excited electronic state to the ground state and geminate or caged recombination of the nascent pentacoordinate species and free NA ligand. Although the latter is unlikely to be affected, the relaxation of excited states following TPA may differ from OPA, given that TPA and OPA have different selection rules which govern the states that are initially populated, and the efficiency of the flow into the ′desirable′ pathway may be lower. If the OPA versus TPA dynamics are comparable, then TPA drug design may be approached by using knowledge garnered from (the more numerous and easily conducted) OPA studies. This study appears to be the first to address this issue, with far‐reaching repercussions in future PDT drug design (vide supra).

**Figure 1:**
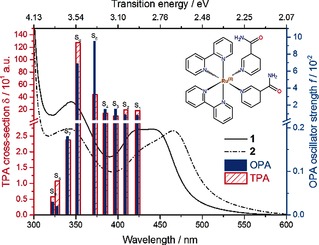
Static UV/Vis absorption spectra of *cis*‐[Ru(bpy)_2_(NA)_2_]^2+^ (solid line, **1**: structure shown) and its mono‐aquated form, *cis*‐[Ru(bpy)_2_(NA)(H_2_O)]^2+^ (dash‐dot line, **2**) formed by irradiation of **1** in water with a blue (465 nm) light source, which yields **2**. DFT calculated OPA oscillator strengths, *f* (blue, right axis) and TPA cross sections, *δ* (red, left axis) for **1** in the gas phase.

We have used transient electronic (UV/Vis) absorption spectroscopy (TEAS) and complementary density functional theory (DFT) calculations to elucidate the excited‐state dynamics of **1** following TPA. For TEAS, a 650 μm aqueous sample of **1** was probed following irradiation with an 800 nm, femtosecond (fs) pulsed laser. The TEAS setup is described elsewhere,^12^, ^13^ and briefly in the Supporting Information. The recorded transient absorption spectra (TAS) for select pump–probe time delays are shown in Figure 2. The initial 5 ps is dominated by a multiphoton signal from the sample cell (glass window), thus our experimental analysis here does not consider the initial excited state evolution. The UV/Vis excitation of this class of Ru^II^ polypyridyl complex (most notably [Ru(bpy)_3_]^2+^) has been heavily studied^14^ and it is typical for the initial excited state to evolve to a manifold of near‐degenerate metal‐to‐ligand charge‐transfer triplet states (^3^MLCT), formed by intersystem crossing from the initially photopopulated (singlet) ^1^MLCT state, with near‐unity quantum yield.^15^ Dissociation may then occur through a triplet metal‐centered (^3^MC, d–d ligand field) state, if it is of a similar energy to the ^3^MLCT.

**Figure 2:**
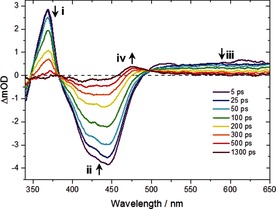
Two‐photon excitation (800 nm) transient absorption spectra of *cis*‐[Ru(bpy)_2_(NA)_2_]^2+^ in water for selected time delays. The features are very similar to those seen following OPA (Figure S2).

To further address the nature of the initially populated excited states, we performed DFT calculations with the CAM‐B3LYP functional for both OPA and TPA. This functional was shown to give accurate TPA transition strengths relative to highly correlated methods,^16^ owing to its ability to better describe transitions to and from intermediate states in a sum‐over‐states representation of the transition tensor.^17^ Further details of the calculations are in the Supporting Information. The one‐ and two‐photon intensities of the first nine singlet‐state transitions are shown in Figure 1. Assessment of the optically bright states (see Table S1 in the Supporting Information) indicates that the dominant contribution of these is of MLCT character, with MC states adding very little to either OPA or TPA intensities. Although direct population of the dissociative state may be possible, that is, ^1^MLCT→^3^MC, little evidence is found for such behavior in the literature.

The TAS shown in Figure 2 comprises three identifiable regions for pump–probe delays of 5–100 ps and an additional feature appearing from 200 ps onwards. A strong negative signal is observed, centered at 420 nm (Figure 2, feature ii), which, as it closely resembles the static UV/Vis absorption of **1** (Figure 1, solid line), is assigned to the ground‐state bleach (GSB) signal. This signal is narrowed significantly, owing to the overlapping of positive signals at approximately 370 and 475 nm. UV/Vis spectroelectrochemistry measurements of [Ru(bpy)_3_]^2+[18]^ and related complexes with functionalized bpy derivatives^19^ indicate that the strong, positive feature at 370 nm (Figure 2, feature i) can be assigned to the excited‐state absorption (ESA) of a ^3^MLCT excited state, and specifically corresponds to an absorption from the bpy anion (bpy^−^) present within the formally charge‐separated character of the ^3^MLCT state (i.e. [Ru^III^(bpy)(bpy^−^)(NA)_2_]^2+^).^20^ The broad plateau of the transient absorption signal at *λ*>550 nm (Figure 2, feature iii) is assigned to a pentacoordinate intermediate (PCI) complex, [Ru(bpy)_2_(NA)]^2+^, in agreement with previously calculated gas‐phase absorption profiles.^12^ Following these features over the first few hundred picoseconds, the GSB feature (ii) recovers back toward zero, whereas the ^3^MLCT ESA feature (i) and the PCI feature (iii) concomitantly decay. As mentioned above, beyond 200 ps, there is a new absorption feature centered at 475 nm (Figure 2, feature iv) that reaches a maximum after 500 ps, which, owing to the resemblance with the static UV/Vis absorption spectrum of **2** (Figure 1, dash‐dot line), can be assigned to the aquation of the PCI and the formation of photoproduct **2**. The photoproduct signal and the corresponding GSB signal remain constant for the remaining probed time delays (up to 2 ns).

Confirmation of a TPA is provided by measuring the dependence of the GSB signal (magnitude of GSB signal at 420 nm for a given pump–probe time delay) with laser excitation power. This is shown in a log–log plot in Figure S3 in the Supporting Information. As there is a second‐order dependence of TPA on the excitation intensity, the gradient of approximately 2 in the linear fit confirms that the excitation is indeed mediated through TPA. From this point on, we seek to gain insight into the TPA activation mechanism and to identify differences for the OPA case by examining kinetic traces for these key features and the timescales involved. Owing to the shrouding of early time dynamics by the glass‐only signal, a full analysis discovering ultrafast (<1 ps) processes cannot be performed. In our previous OPA study (excitation at 340 nm),^12^ where such time resolution was available, a detailed ′target analysis′ approach was used to extract time constants and quantum yields for the branched kinetics, as well as to formulate the general mechanism shown in Figure 3. The time constants extracted from the following analysis are used to qualitatively show that the photoactivation of **1** following TPA proceeds along the same (if not, very similar) pathway as that following OPA, that is, **1**+*hν*→→ ^3^MLCT→ ^3^MC→ PCI→ **2**.

**Figure 3:**
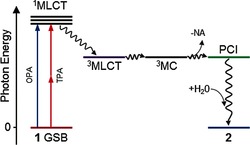
Schematic for the photoactivation mechanism of **1**. Following excitation with either OPA or TPA of energy equivalent to approximately 3–4 eV, **2** is formed through a dissociative ^3^MC state and five‐coordinate intermediary state. Nonreactive pathways are omitted. Colored states correspond to features in the TAS that are used in the kinetic analysis (see Figure 4): GSB of **1**, red, feature ii; ^3^MLCT, violet, feature i; **2**, blue, feature iii; and PCI, green, feature iv.

The target analysis uses basis functions derived from known absorption profiles, fitted to spectra at each pump–probe time delay, to extract an integrated signal for each spectral component over time. These transients are shown in Figure 4 (with corresponding OPA traces shown in Figure S4 in the Supporting Information). Fitting of mono‐exponential decay functions yields time constants for the evolution of the ^3^MLCT, PCI, **2**, and GSB recovery. It is important to note that, for a branched kinetics scheme, where there are competing relaxation pathways for these features, for example, the ^3^MLCT state may convert to either the ground state or ^3^MC state, there are at least two lifetimes contributing to the time constant extracted from a mono‐exponential fit. The fits returned the time constants shown in Table 1 for the evolution of features i, ii, iii, and iv (Figures 4 a–d, respectively), which were 188±2 ps for the ^3^MLCT state, 198±4 ps for the GSB recovery, 167±7 ps for the PCI species, and 95±5 ps for formation of **2**. These time constants compare favorably with the time constants determined by using the same analysis of one‐photon excitation data (see Figure S4 in the Supporting Information) with the exception of *k*_**2**_. This is unsurprising given the strong overlap between the spectral features of **2** and all other features here, and that the population (amplitude) of this signal is determined at an early time (<5 ps), so that we do not fully resolve for our TPA data (as was done with the OPA experiment).

**Figure 4:**
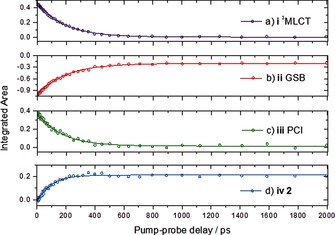
Kinetic traces for time‐dependent evolution (following excitation with two photons of 800 nm) of a) ^3^MLCT (i) state population, b) ground‐state bleach (ii) recovery, c) PCI (iii) population, and d) **2** the photoproduct (iv), obtained from integration of basis functions used in the ′target analysis′. The obtained lifetimes are summarized in Table 1.

**Table 1:** Time constants [ps] obtained from the kinetic analysis.

	^3^MLCT	GSB	PCI	**2**
OPA	183±2	179±3	168±9	151±9
TPA	188±2	198±4	167±7	95±5

The general agreement of these time constants suggests that the dynamics following either TPA or OPA follow the same pathways and timescales, that is, it is only the initially populated state that is different for either absorption, and the population still arrives at the ^3^MLCT state following intersystem crossing. To assert this fully, a complete kinetic analysis using fitting functions derived from the branched kinetic scheme (not simple exponentials) and simultaneous fitting would be needed. For the previously published OPA data, it was possible to evaluate all quantum yields for branching of reaction pathways by using target analysis and simultaneously fitting traces with multi‐exponential functions derived from a full kinetic scheme. Owing to the lack of early time data (<5 ps) for TPA, we are unable to elucidate critical ultrafast timescales that affect the evolution of the initially excited states and branching ratios. Certainly, we cannot fully rule out other deactivation pathways that compete with intersystem crossing of the ^1^MLCT to ^3^MLCT, for example, ^1^MLCT→^3^MC→GS.^21^ However, we can still compare the final GSB recovery of both OPA and TPA. Doing so reveals that the GSB recovery after TPA is approximately equal to that of the OPA, suggesting that, for the TPA formation of **2**, this returns a quantum yield of *Φ*≈0.4 (based on previous OPA analysis).

To summarize, we have demonstrated for the first time that the evolution of a photoactivatable prodrug following two‐photon activation can be probed. Surprisingly, TPA *and* OPA produce the same photochemistry. The fact that the TPA and OPA mechanisms are the same (or at the very least, very similar)—attributed here to “funneling” of excited‐state flux into the ^3^MLCT manifold—means that it may be possible to alter the structure of **1** and repeat OPA measurements with the intention of employing TPA in a final clinical stage. This “bottom‐up approach” to study and tune the OPA is more easily achieved, owing to the higher absorption strengths and the experimental simplicity that this provides. Further studies, in which the glass response is reduced to allow for the study of the very early dynamics, may, however, prove lucrative. This could be achieved through the use of molecules with larger TPA cross sections or glass‐free sample delivery, such as with a thin‐film liquid jet. Additionally, photoexcitation to higher energies, where the effects of different selection rules for OPA versus TPA are likely to be more pronounced (owing to the increased density of states) may result in different excited‐state dynamics and could provide interesting results, in particular if competing pathways other than the main MLCT→GS relaxation are identifiable. We conclude by noting that the photophysics of Ru^II^ complexes is well studied and as such provides a fruitful base from which other similar complexes may be designed. Particularly, the use of ligands that increase the TPA cross section will prove critical if this type of species is to find clinical use. Such complexes have been made and are now being studied by using OPA TEAS to evaluate their efficacy.

## Supporting information

As a service to our authors and readers, this journal provides supporting information supplied by the authors. Such materials are peer reviewed and may be re‐organized for online delivery, but are not copy‐edited or typeset. Technical support issues arising from supporting information (other than missing files) should be addressed to the authors.

Supplementary
