# Supplementary material for: Excited‐State Dynamics of a Two‐Photon‐Activatable Ruthenium Prodrug
Source: Chemphyschem. 2016 Jan 6;17(2):221–4. doi: 10.1002/cphc.201501075 (PMC4797363; doi:10.1002/cphc.201501075)
Supplement: Supplementary file 1 — Supplementary [file CPHC-17-221-s001.pdf]

CHEMPHYSCHEM

Supporting Information

Excited-State Dynamics of a Two-Photon-Activatable Ruthenium Prodrug

Simon E. Greenough,[a, c] Michael D. Horbury,[a] Nichola A. Smith,[a] Peter J. Sadler,[a] Martin J. Paterson,[b] and Vasilios G. Stavros*[a]

cphc_201501075_sm_miscellaneous_information.pdf

Supporting Information

Experimental Methods

Static UV/visable absorption spectra

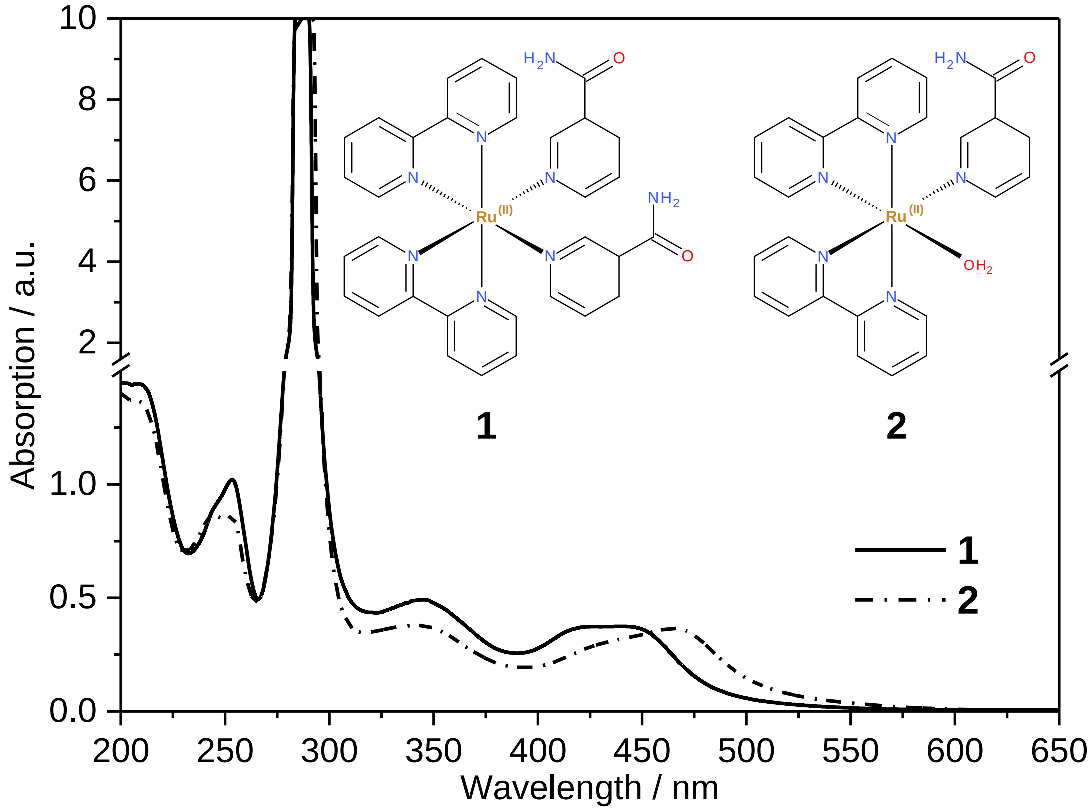

Figure S1 Aqueous UV/Vis absorption spectra and structures of $Ru[(bpy)_2(NA)_2]^{2+}$ (**1**) and $Ru[(bpy)_2(NA)(H_2O)]^{2+}$ (**2**).

Transient UV/visible (electronic) absorption spectroscopy (TEAS)

650 µM aqueous samples of $Ru[(bpy)_2(NA)_2]^{2+}$ (**1**) were delivered using a steel flow-through cell (Harrick Scientific), comprising two CaF_2 windows and a 950 µm thick Teflon spacer, which defines the optical path length. The sample was recirculated using a peristaltic pump (Masterflex) with PTFE tubing throughout, at a flow speed sufficient to ensure fresh solution was sampled with each laser shot. Sample was excited using 800 nm (1.55 eV), ~50 fs pump pulses. The pump beam was focused ~20 mm behind the sample to ensure a beam waist at the sample of ~250 µm (~5 times that of the probe) and return pump fluences of ~20 mJ cm^{-2}. The use of a 500 Hz mechanical chopper (Thorlabs) in the pump beam creates an alternating pumped and non-pumped sample from which a difference spectrum may be calculated after probing. Pump–probe delays (up to 2 ns) are created using a motorized optical delay line in the probe beam path.

Pump and probe pulses are generated from a commercially available femtosecond Ti-sapphire regenerative amplified laser system (SpectraPhysics, Spitfire XP) operating at 1 kHz. The output the laser system is split to give two 800 nm beams: (i) 950 mW and (ii) 5 mW. (i) is attenuated to provide a pump power of 24 mW (this maximum power limit prevents white light generation in the sample). Broadband white light continuum (340 to 675 nm) probe pulses are generated by focusing an attenuated (ii) into a vertically translated CaF_2 window and detected using a fibre couple UV/Vis spectrometer (Avantes, AvaSpec Ultrafast). A purpose built LabVIEW code controls the system and acquires data.

Previously reported one-photon absorption (OPA) TEAS spectra

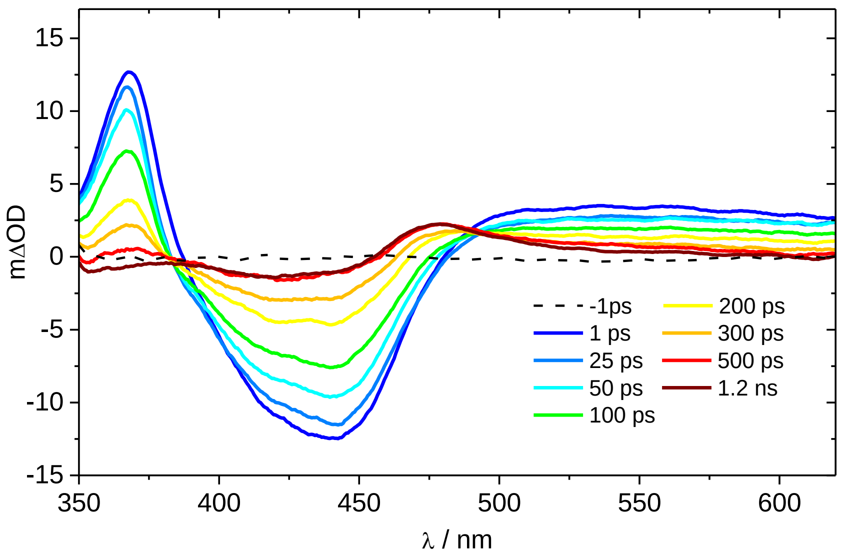

Figure S2 OPA excitation (340 nm) transient absorption spectra of *cis*-$[Ru(bpy)_2(NA)_2]^{2+}$ in water for select pump-probe time delays.[1]

Power dependence

To confirm that the excitation of **1** at 800 nm is a two-photon process, spectra were recorded at select time delays at five different excitation powers 12, 15, 18, 21 and 24 mW corresponding to excitation fluences of between ~10 and ~20 mJ cm^{-2}. Figure S3 shows the log-log plot of the ground state bleach (GSB) intensity for a pump-probe delay of 100 ps (taken from the average of 10 pixels around 420 nm) against excitation power. The gradient in the linear fit of ~2 is a clear indicator of second order dependence, and hence confirms that two-photon absorption (TPA) is responsible for the observed signal.

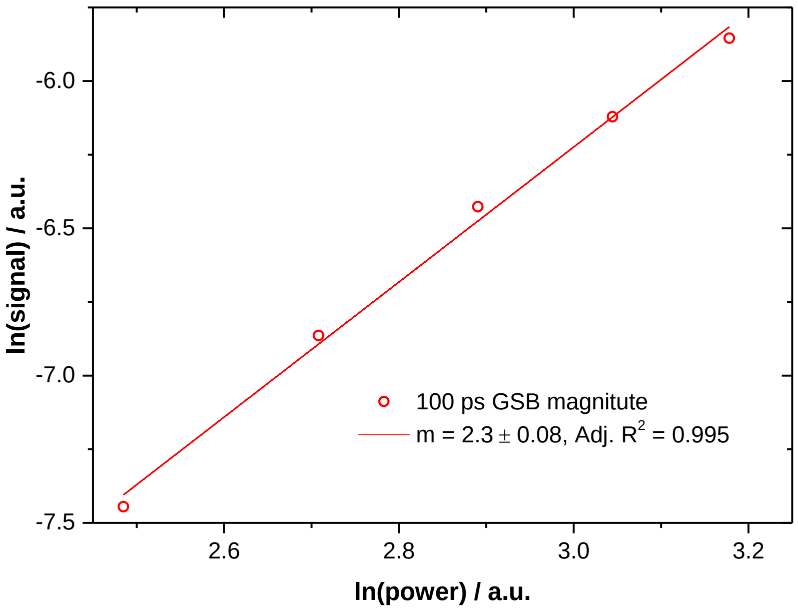

Figure S3 Log-log plot of ground state bleach signal at 100 ps against excitation power.

Comuptational Details

The ground state geometry of **1** was optimized using density functional theory (DFT). Analytical Hessian evaluation confirmed the nature of the optimized geometries as minima. Following our earlier work[1] testing a variety of functionals and basis sets on this and similar systems (including the use of non-, quasi-, and fully relativistic effective core potentials), we have used M06L functional in conjunction with a SDD effective-core potential (ECP) for ruthenium: 28 core electrons modelled on neutral ruthenium with a Wood-Boring quasi-relativistic potential, together with the cc-pVTZ valence set of orbitals for both ruthenium and other atoms (cc-pVTZ from Ref. [2] for ruthenium with an ECP).

The CAM-B3LYP functional was used for two-photon absorption calculations. This has been shown to give accurate TPA transition-strengths relative to highly correlated methods,[3] due to its ability to better describe transitions to and from intermediate states in a sum-over-states representation of the transition tensor.[4]

For OPA and TPA the one-electron basis was similar to that discussed above, but made less computationally expensive, namely: the same ECP for ruthenium, but the standard SDD double-zeta valence basis set for ruthenium,[5] and the 6-311G(d) basis for other atoms. For OPA this basis gave a small, consistent 0.1-0.2 eV red-shift compared to the larger one. We note that B3LYP generally gives excitation energies 0.5-0.6 eV lower than CAM-B3LYP for both notionally MLCT and LF states.

To investigate the effect of solvent on excitation energies a polarizable water continuum was employed. In general this causes a maximum increase in excitation energies of ~0.1 eV, although for most states the effect is even less.

One- and two-photon absorption were calculated from the linear and quadratic response functions,[6] the poles giving excitation energies while the residues give transition moments. For one-photon absorption the transition probability is presented as the oscillator strength,

$$f = \sum_{\alpha} \tfrac{2}{3}\omega_{if}\left|\langle i|\mu_{\alpha}|f\rangle\right|^{2}$$

where ω_{ij} is the excitation energy from state $|i\rangle$ to state $|j\rangle$, with μ_{α} the Cartesian components of the electric dipole operator, and all quantities expressed in atomic units. For TPA the transition tensor (S) is obtained from the first residue of the quadratic response function.[7] This can be written in a sum-over-states spectral representation as,[8]

$$S_{\alpha\beta} = \sum_{j} \frac{\langle i|\mu_{\alpha}|j\rangle\langle j|\mu_{\beta}|f\rangle}{\omega_{j}-\omega} + \frac{\langle i|\mu_{\beta}|j\rangle\langle j|\mu_{\alpha}|f\rangle}{\omega_{j}-\omega}$$

where ω_j denotes the excitation frequency of the jth state relative to the ground state, ω the frequency of the irradiating light, and μ_{α} and μ_{β} are the spatially-dependent components of the electric dipole operator. The two-photon calculations were carried resonant photons each with energy equal to half the vertical excitation energy of the final state. The calculated two-photon transition strength (in atomic units), δ^{TPA}, is reported as a rotationally averaged quantity, defined as,[9]

$$\delta^{TPA} = F\delta^{F} + G\delta^{G} + H\delta^{H}$$

$$\delta^{F} = \frac{1}{30}\sum_{\alpha,\beta} S_{\alpha\alpha}S^{*}_{\beta\beta}$$

$$\delta^{G} = \frac{1}{30}\sum_{\alpha,\beta} S_{\alpha\beta}S^{*}_{\alpha\beta}$$

$$\delta^{H} = \frac{1}{30}\sum_{\alpha,\beta} S_{\alpha\beta}S^{*}_{\beta\alpha}$$

where the summations are performed over the molecular axes (*i.e.*, *x*, *y*, and *z* in Cartesian coordinates), and *F*, *G* and *H* depend on the polarization of the incident photons. Under conditions in which excitation is achieved using linearly polarized light, $F = G = H = 2$, as applied here.

Geometry optimization was performed using the Gaussian09 program,[10] while one- and two-photon absorption were calculated with the Dalton2015 program.[11]

Table S1 Calculated CAM-B3LYP transition energies, oscillator strengths, *f*, and two photon absorption cross-sections, δ, for the first 11 singlet states of complex **1** in the gas phase.

State	Energy / eV	λ / nm	f	δ^{TPA}	Character	Dom. Contribution
S_1	2.93	423	0.0080	182.0	MLCT	78% H→ L
S_2	3.03	409	0.0088	196.0	MLCT	77% H → L+1
S_3	3.12	397	0.0154	94.6	MLCT	57% H-1 → L<br>29% H-1 → L+1
S_4	3.22	385	0.0152	138.0	MLCT	59% H-1 → L+1<br>23% H-1 → L
S_5	3.33	372	0.0952	442.0	MLCT	86% H-2 → L
S_6	3.52	352	0.0688	1270.0	MLCT	70% H-2 → L+1
S_7	3.64	341	0.0018	23.7	LF	43% H→ L+16
S_8	3.78	328	0.0002	10.7	LF	23% H-2→ L+15
S_9	3.84	323	0.0003	5.8	LF	30% H-1→ L+15
S_{10}	4.17	297	0.0311	812.0	MLCT	44% H → L+3
S_{11}	4.22	294	0.0090	95.3	MLCT	57% H → L+2

Table S2 Calculated CAM-B3LYP transition energies for the first 10 triplet states of complex **1** in the gas phase.

State	Energy / eV	λ / nm	Character	Dom. Contribution
T_1	2.7049	458	MLCT	29% H→ L
T_2	2.7546	450	MLCT	27% H→ L+1<br>22% H-5→ L+1
T_3	2.9098	426	MLCT	33% H-1 → L
T_4	3.0150	411	MLCT	38% H-1 → L+1
T_5	3.1526	393	LF	44% H → L+16
T_6	3.2318	384	LF	21% H → L+15
T_7	3.2666	380	mixed	27% H-2 → L
T_8	3.3272	373	LF	27% H-1 → L+15
T_9	3.3574	369	MLCT	23% H-2 → L+1
T_{10}	3.3860	366	MLCT	27% H-1 → L

Kinetic analysis

Kinetic traces for each of the individual species involved in the photodissociation of **1** were extracted using a target analysis approach. This was achieved using the recently developed kinetics analysis software package KOALA. For further details on the exact methods used in this fitting program the reader is referred to the comprehensive description of KOALA provided in Ref. [12] and the Supporting Information of Ref. [1]. The resulting kinetic trace for OPA is shown in Figure S4.

OPA kinetic traces

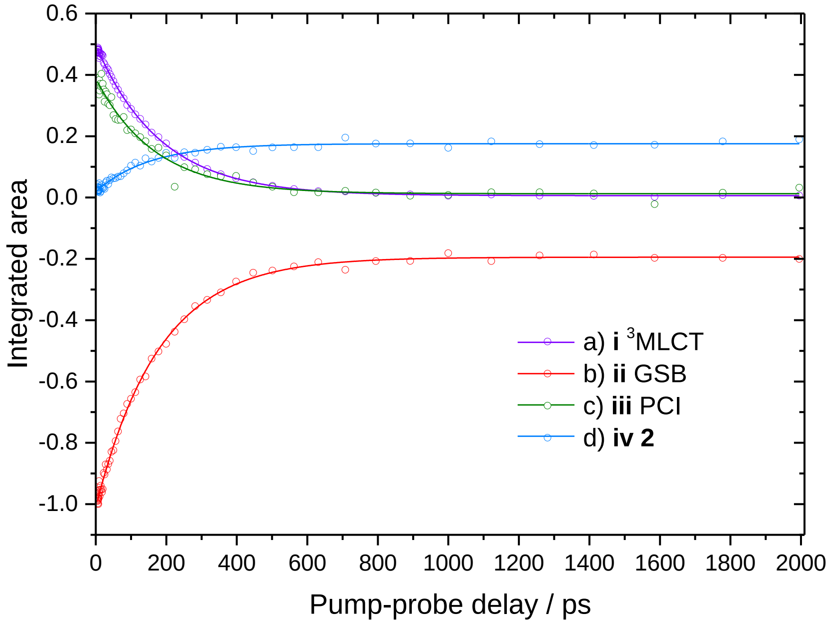

Figure S4 Kinetic traces for time-dependent evolution (following 340 nm excitation) of a) ^{3}MLCT (**i**) state population, b) ground state bleach (**ii**) recovery, c) PCI (**iii**) population, and d) **2** the photoproduct (**iv**), obtained from integration of basis functions used in the 'target analysis'.

References

[1] S. E. Greenough, G. M. Roberts, N. A. Smith, M. D. Horbury, R. G. McKinlay, J. M. Zurek, M. J. Paterson, P. J. Sadler, V. G. Stavros, *Phys. Chem. Chem. Phys.* **2014**, *16*, 19141-19155.

[2] K. A. Peterson, D. Figgen, M. Dolg, H. Stoll, *J. Chem. Phys.* **2007**, *126*, 124101.

[3] M. J. Paterson, O. Christiansen, F. Pawłowski, P. Jørgensen, C. Hättig, T. Helgaker, P. Sałek, *J. Chem. Phys.* **2006**, *124*, 054322.

[4] a) J. Arnbjerg, M. J. Paterson, C. B. Nielsen, M. Jørgensen, O. Christiansen, P. R. Ogilby, *J. Phys. Chem. A* **2007**, *111*, 5756-5767; b) L. T. Bergendahl, M. J. Paterson, *J. Phys. Chem. B* **2012**, *116*, 11818-11828.

[5] D. Andrae, U. Häußermann, M. Dolg, H. Stoll, H. Preuß, *Theor. Chim. Acta* **1990**, *77*, 123-141.

[6] O. Christiansen, P. Jørgensen, C. Hättig, *Int. J. Quantum Chem* **1998**, *68*, 1-52.

[7] J. Olsen, P. Jo/rgensen, *J. Chem. Phys.* **1985**, *82*, 3235-3264.

[8] T. D. Poulsen, P. K. Frederiksen, M. Jørgensen, K. V. Mikkelsen, P. R. Ogilby, *J. Phys. Chem. A* **2001**, *105*, 11488-11495.

[9] a) P. R. Monson, W. M. McClain, *J. Chem. Phys.* **1970**, *53*, 29-37; b) W. M. McClain, *J. Chem. Phys.* **1971**, *55*, 2789-2796.

[10] Gaussian 09, Revision A.02, Frisch, M. J.; Trucks, G. W.; Schlegel, H. B.; Scuseria, G. E.; Robb, M. A.; Cheeseman, J. R.; Scalmani, G.; Barone, V.; Mennucci, B.; Petersson, G. A.; Nakatsuji, H.; Caricato, M.; Li, X.; Hratchian, H. P.; Izmaylov, A. F.; Bloino, J.; Zheng, G.; Sonnenberg, J. L.; Hada, M.; Ehara, M.; Toyota, K.; Fukuda, R.; Hasegawa, J.; Ishida, M.; Nakajima, T.; Honda, Y.; Kitao, O.; Nakai, H.; Vreven, T.; Montgomery, J. A., Jr.; Peralta, J. E.; Ogliaro, F.; Bearpark, M.; Heyd, J. J.; Brothers, E.; Kudin, K. N.; Staroverov, V. N.; Kobayashi, R.; Normand, J.; Raghavachari, K.; Rendell, A.; Burant, J. C.; Iyengar, S. S.; Tomasi, J.; Cossi, M.; Rega, N.; Millam, N. J.; Klene, M.; Knox, J. E.; Cross, J. B.; Bakken, V.; Adamo, C.; Jaramillo, J.; Gomperts, R.; Stratmann, R. E.; Yazyev, O.; Austin, A. J.; Cammi, R.; Pomelli, C.; Ochterski, J. W.; Martin, R. L.; Morokuma, K.; Zakrzewski, V. G.; Voth, G.

A.; Salvador, P.; Dannenberg, J. J.; Dapprich, S.; Daniels, A. D.; Farkas, Ö.; Foresman, J. B.; Ortiz, J. V.; Cioslowski, J.; Fox, D. J.; Gaussian, Inc., Wallingford CT, 2009

[11] K. Aidas, C. Angeli, K. L. Bak, V. Bakken, R. Bast, L. Boman, O. Christiansen, R. Cimiraglia, S. Coriani, P. Dahle, E. K. Dalskov, U. Ekström, T. Enevoldsen, J. J. Eriksen, P. Ettenhuber, B. Fernández, L. Ferrighi, H. Fliegl, L. Frediani, K. Hald, A. Halkier, C. Hättig, H. Heiberg, T. Helgaker, A. C. Hennum, H. Hettema, E. Hjertenæs, S. Høst, I.-M. Høyvik, M. F. Iozzi, B. Jansik, H. J. Aa. Jensen, D. Jonsson, P. Jørgensen, J. Kauczor, S. Kirpekar, T. Kjærgaard, W. Klopper, S. Knecht, R. Kobayashi, H. Koch, J. Kongsted, A. Krapp, K. Kristensen, A. Ligabue, O. B. Lutnæs, J. I. Melo, K. V. Mikkelsen, R. H. Myhre, C. Neiss, C. B. Nielsen, P. Norman, J. Olsen, J. M. H. Olsen, A. Osted, M. J. Packer, F. Pawlowski, T. B. Pedersen, P. F. Provasi, S. Reine, Z. Rinkevicius, T. A. Ruden, K. Ruud, V. Rybkin, P. Salek, C. C. M. Samson, A. Sánchez de Merás, T. Saue, S. P. A. Sauer, B. Schimmelpfennig, K. Sneskov, A. H. Steindal, K. O. Sylvester-Hvid, P. R. Taylor, A. M. Teale, E. I. Tellgren, D. P. Tew, A. J. Thorvaldsen, L. Thøgersen, O. Vahtras, M. A. Watson, D. J. D. Wilson, M. Ziolkowski, and H. Ågren, "The Dalton quantum chemistry program system", WIREs Comput. Mol. Sci. 2014, 4:269–284.

[12] M. P. Grubb, A. J. Orr-Ewing, M. N. Ashfold, *Rev. Sci. Instrum.* **2014**, *85*, 064104.
